# Gene Expression during Survival of *Escherichia coli* O157:H7 in Soil and Water

**DOI:** 10.1155/2011/340506

**Published:** 2010-09-29

**Authors:** Ashley D. Duffitt, Robert T. Reber, Andrew Whipple, Christian Chauret

**Affiliations:** ^1^Earth and Environmental Sciences, Taylor University, Upland, IN 46989, USA; ^2^Department of Natural, Information, and Mathematical Sciences, IN University Kokomo, 2300 South Washington Street, Kokomo, Indiana 46904-9003, USA; ^3^Department of Biology, Taylor University, Upland, IN 46989, USA

## Abstract

The *in vitro* survival of *Escherichia coli* O157:H7 at 15°C under two experimental conditions (sterile soil and sterile natural water) was examined. DNA microarrays of the entire set of *E. coli* O157:H7 genes were used to measure the genomic expression patterns after 14 days. Although the populations declined, some *E. coli* O157:H7 cells survived in sterile stream water up to 234 days and in sterile soil for up to 179 days. Cells incubated in soil microcosms for 14 days expressed genes for antibiotic resistance, biosynthesis, DNA replication and modification, metabolism, phages, transposons, plasmids, pathogenesis and virulence, antibiotic resistance, ribosomal proteins, the stress response, transcription, translation, and transport and binding proteins at significantly higher levels than cells grown in Luria broth. These results suggest that *E. coli* O157:H7 may develop a different phenotype during transport through the environment. Furthermore, this pathogen may become more resistant to antibiotics making subsequent infections more difficult to treat.

## 1. Introduction

*Escherichia coli *O157:H7 is an enterohemorrhagic strain of * E*. *coli* that produces a powerful shiga-like toxin. It is capable of causing bloody stools, hemorrhagic colitis, and hemolytic uremic syndrome [1]. Nearly 75,000 cases of O157:H7 infection occur every year in the US [2]. Most outbreaks have been associated with the consumption of contaminated, undercooked, bovine food products [1]. There also have been reports of *E*. *coli *O157:H7 outbreaks associated with both drinking and recreational water [3–7].

*E*. *coli *O157:H7 is ubiquitous on farms where healthy cattle and sheep harbor the pathogen in their gastrointestinal tracts [8]. As a consequence, farm animal manure is a source for spreading *E*. *coli* O157:H7 into the environment and potentially to the human food chain. One of the most common modes by which *E*. *coli *O157:H7 is introduced onto food crops is through contaminated irrigation water [9]. In addition, the propagation of this pathogen through the environment has been linked to runoff contaminated with bovine manure or by use as soil amendment [10]. The contamination of surface and ground water in rural areas of the United States is becoming increasingly more common as a result of concentrated animal feeding operations [9]. 

*Escherichia coli *O157:H7 can survive in varying habitats under a wide range of conditions. In the environment, cells are exposed to rapidly changing conditions such as changes in pH, nutrient availability, temperature, oxidative stress, and osmotic challenge [11]. *E*. *coli *O157:H7 pollution of water and soil is dependent on the ability of this pathogen to adapt to these changes. However, there is limited information concerning the survival of *E*. *coli *O157:H7 in soil and water as growth of bacteria, if any, in these environments is not well understood or documented. *E*. *coli* O157:H7 may respond to adverse conditions in the environment by expressing various stress response genes that enable survival [12]. The master regulator of the general stress response is an alternative sigma factor *σ* ^38^ (RpoS). This sigma factor may be induced in response to stresses such as weak acids, starvation, high osmolarity, and high or low temperature [13]. There is evidence that stress responses may enable survival under more severe conditions, increase pathogenicity, and enhance resistance to secondary stresses [14]. In other words, when *E*. *coli* cells are stressed, they become harder to kill and are more resistant to starvation and toxic chemicals typically used in distribution systems such as chlorine. This has significant public health implications because *E*. *coli *O157:H7 could develop a disinfectant-resistant phenotype during transport to water treatment plants [14]. Therefore, understanding the effects of stress on gene expression in response to altered environmental conditions may be crucial in understanding the survival of this organism as it moves from one environment to another. 

Limited work has been conducted to investigate *E*. *coli *O157:H7 survival and functional genomics in the environment. Further research is needed to understand the mechanisms that enable *E*. *coli* O157:H7 to survive in such a wide variety of environments. This study compared genetic expression profiles of *Escherichia coli* O157:H7 under two environmental conditions (soil and natural water) to expression in growth media using DNA microarrays. In addition, we investigated the long-term survival of *E*. *coli *O157:H7 in microcosms simulating these environments.

## 2. Materials and Methods

### 2.1. Soil and Water Collection and Site Description

Soil and water were collected from Newby Ditch in June of 2006 within 24 hours of a rainfall event (1.93 cm or 0.76 inches). Newby Ditch is located within the Mississinewa watershed on the Tipton Till Plain of East Central Indiana (USA). This watershed is characterized as a highly disturbed landscape predominated by row crop agriculture. Environmental samples were placed in sterile bottles and stored in a cooler with ice packs for transport to the lab. The samples were processed within 24 hours for use as soil and water microcosms according to the methods described below.

### 2.2. Inoculum and Incubation Conditions

*E*. *coli *O157:H7 strain ATCC 35150 was used to investigate survival and differences in gene expression under two different environmental conditions compared to Luria broth (LB) (BD, Rockville, MD). For long-term storage, the culture was maintained in 10% glycerol at −50°C. In the short term, cells were cultured on nutrient agar (BD) slants at 4°C. The *E*. *coli *O157:H7 experiment cultures were prepared by transferring a loopful of a slant culture into a 100 mL flask containing 30 mL of LB. The flask was incubated for 24 hours at 37°C without shaking. This culture was used to inoculate the three treatment conditions: 30 mL LB control, 90 mL sterile stream water microcosms, and 100-g sterile soil microcosms. The 30 mL LB control (100 mL flask) was inoculated with 1 mL of the culture and incubated at 15°C for 48 hours with shaking at 60 rpm. The remaining cells were harvested by centrifugation at 10,000 × g for 10 minutes at 4°C (Sorvall RC-5B), washed twice with sterile deionized water, and resuspended in 10 mL of sterile stream water or 10 mL of sterile deionized water. This suspension was used to inoculate the stream water and soil microcosms, respectively. The control and water experiments were performed using ten replicates each, four for microarray analysis and six to monitor survival with replacement. The soil treatment was prepared with 34 replicates, four for microarray analysis and 30 to monitor survival without replacement. The water microcosms were incubated at 15°C with shaking at 60 rpm, and the soil microcosms were incubated at 15°C without shaking. Four microcosms, each of soil and water, were removed after 14 days for DNA microarray analysis. Four flasks of the 15°C LB control were removed after 48 hours for DNA microarray analysis. Survival was assessed periodically using the spread plate method described below.

### 2.3. Water Microcosms

The stream water used in this experiment had a pH of 7.26. Aliquots of water (90 mL) were placed into 250 mL sterile bottles and autoclaved at 121°C at 15 psi for 15 minutes. Following sterilization, the water microcosms were stored at 4°C until inoculation. Following inoculation with 8.8 ×10^8^ CFU/mL of *E*. *coli *O157:H7 ATCC 35150, the water microcosms were incubated at 15°C with shaking at 60 rpm.

### 2.4. Soil Microcosms

The soil used in this study was a Fox silt loam with a pH of 6.95 [15]. Fox silt loam is a typical agricultural soil in East Central Indiana. It is a well-drained soil with a moderate available water capacity, medium runoff, and poor filtering capacity [15]. Soil pH was determined by placing 20 g of soil into a 50 mL beaker, adding 20 mL of deionized water, and stirring for 30 minutes [16]. This suspension was allowed to settle for an hour, and pH was measured with a pH meter. Soil moisture content was determined using the gravimetric method; soil was weighed, oven dried at 105°C, and then reweighed until the sample weight was constant [16]. The soil moisture content was determined to be 0.323 (32.3%), which is a slightly moist soil [15]. Large rocks and debris were removed from the dried soil, and 100-g aliquots were placed in 250 mL glass bottles. The soil microcosms were autoclaved at 121°C at 15 psi for 15 minutes Following sterilization, the soil was returned to the drying oven for 24 hours at 105°C to remove any residual moisture. Sterile soil, as checked by plate counting, was stored at room temperature prior to inoculation. Soil moisture content was adjusted to field conditions by adding 32.3 mL of sterile deionized water [16]. Following inoculation with 8.8 × 10^8^ CFU/ml of *E*. *coli *O157:H7 ATCC 35150, the microcosms were sealed and incubated statically at 15°C.

### 2.5. Colony Counting

The initial (zero-time postinoculation) concentration *E*. *coli *O157:H7 was determined for each condition prior to incubation. *E*. *coli *O157:H7 concentrations were determined for each condition by using the spread plate method. Samples for counting were removed directly from the water microcosms and LB broth. For extraction of bacteria from soil, 100 mL 0.1% polyethylene glycol (PEG) was added to 100 g of soil. This suspension was shaken (150 rpm) for 15 minutes at 15°C and then allowed to settle for five minutes. The supernatant from this suspension was centrifuged for ten minutes (10,000 × g, 4°C), and the resulting supernatant was used to enumerate the cells. Viable cell counts were performed in duplicate by serial dilution in 0.9% sterile saline and spread plate culturing 0.1 mL onto R2A agar (BD) plates. The plates were incubated at 37°C for 48 hours. Following incubation, plates with colony counts between 25 and 250 were considered, and duplicate counts were averaged. The average number of colonies was divided by the volume or mass of the original solution to estimate the number of CFU/ml and CFU/g, respectively.

### 2.6. Isolation of Total RNA

Total RNA was isolated using the FastRNA Pro Soil-Direct Kit (Qbiogene, Inc., CA) with minor modifications (described below) to improve quality and yield. The kit-supplied Lysing Matrix E tubes were placed at −10°C for 2 days prior to RNA extraction to minimize heating and RNA degradation. One gram of the sample was placed into the kit-supplied Lysing Matrix E tubes for lysis. Sample lysis was performed using a Mini-BeadBeater-1 instrument (Biospec Products, Inc., Bartlesville, OK) for 80 seconds at a speed setting of 48 (maximum speed). RNA was centrifuged through the kit-supplied Quick-Clean Spin Filters to remove residual inhibitors following extraction and stored in 100 *μ*L of DEPC-H_2_0 at −50°C. RNA quality was determined by 1.0% (w/v) agarose gel electrophoresis and by spectrophotometric analysis (OD_260_/OD_280_) using a Thermo Scientific Spectronic GENESYS 5 Spectrophotometer. An OD measurement at 260 nm was used to quantify the RNA yield. Approximately 100 *μ*g of total RNA was obtained per sample. The purified RNA samples were delivered on ice to Dr. Howard Edenberg's laboratory at the Indiana University School of Medicine Center for Medical Genomics where the microarray analysis was performed.

### 2.7. DNA Microarrays, cDNA Preparation, and Hybridization

DNA microarrays were used to evaluate the genetic expression profiles of *E*. *coli *O157:H7 ATCC 35150 maintained under three environmental conditions. The microarray system used for *E*. *coli *was the GeneChip *E*. *coli *Genome 2.0 Array (Affymetrix, Inc., Santa Clara, CA). The microarray analysis was performed with four biologically-independent replicates (with respect to *E*. *coli *growth, RNA isolation, sample preparation, and array hybridization) for each treatment condition. 

The standard protocol for prokaryotic sample and array processing recommended by Affymetrix in their GeneChip Expression Analysis Technical Manual (Affymetrix, Santa Clara, CA) was used. cDNA was synthesized using a T7 promoter-dT24 oligonucleotide as a primer with the Invitrogen Life Technologies SuperScrip Choice system. Following second-strand cDNA synthesis and incubation with T4 DNA polymerase, the products were purified using the Affymetrix Cleanup Module. Biotinylated cRNA was made using the Affymetrix IVT kit. The cRNA was purified using the Qiagen RNeasy column, quantitated, and then fragmented by incubation at high temperature with magnesium. Biotinylated cRNA was then added to a hybridization solution and hybridized to the GeneChip after adding control oligonucleotides at 45°C for 17 hours with constant rotation. The hybridization mixture was removed, and the GeneChip was washed and stained with phycoerythrin-labeled Streptavidin using the Affymetrix Fluidics Station. The GeneChip was washed again, incubated with biotinylated antistreptavidin, and then restained with phycoerythrin-labeled Streptavidin to amplify the signals. Balanced groups of samples were handled in parallel to reduce nonrandom error. The arrays were scanned using the dedicated scanner controlled by Affymetrix GCOS software.

### 2.8. Data Analysis

The microarray expression data were generated using Affymetrix GCOS software. The Affymetrix Microarray Suite Algorithm was used to analyze the hybridization intensity data from GeneChip expression probe arrays and to calculate a set of metrics to describe probe set performance. The average intensity of each array was normalized by global scaling to a target intensity of 1000. An average expression value for each treatment group was calculated via geometric mean because it is better applied to data with large fluctuations. Only probe sets that received a “present” call of 75% or greater were considered. The expression values were normalized by log _2_ transformation [17]. Two treatments were compared by determining the log _2_ ratio of gene expression for the corresponding averaged intensities for each treatment. Fold change was calculated from log _2_ data such that values for induction range from 1 to 100 while values for repression are restricted to the space between 0 and 1 [18]. For example, a value of 2 indicates a 2-fold upregulation while a value of 0.5 indicates a 2-fold downregulation for a gene comparing the environmental condition to the Luria broth. A *t*-test on the log _2_ transformed data was performed using Microsoft Excel. Significant gene selection was performed using the Microsoft Excel filter function to select for genes with greater than or equal to a 2-fold up- or downregulation and a *P*-value less than .05.

### 2.9. Functional Groups

 Functions of significantly expressed genes were determined using the Affymetrixs NetAffx Analysis Center (http://www.affymetrix.com/analysis/netaffx/index.affx) and EcoCyc (http://ecocyc.org/) database. Functional group analysis was performed by assigning genes to one of 13 functional groups.

## 3. Results

### 3.1. Survival of *E. coli* O157:H7 in Sterile Soil Microcosms

**Figure 1:**
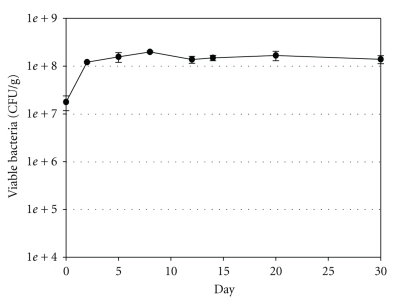
Survival of *E*. *coli *O157:H7 in sterile soil microcosms at 15°C. The average concentrations of *E*. *coli *O157:H7 were determined by the spread plate method and are shown as CFU per gram. Error bars represent standard deviations about the means (*n* = 4).

Soil microcosms were inoculated with 10 mL of 8.8 × 10^8^ CFU/g of *E*. *coli *O157:H7 strain ATCC 35150. Immediately after inoculation, the average preincubation concentration of *E*. *coli *O157:H7 was 1.8 × 10^7^ CFU/g. Following incubation at 15°C, survival was monitored on a regular basis for 179 days. No significant decrease in the *E*. *coli *O157:H7 concentration was observed during the first 30 days of incubation (Figure 1). The last measurement was taken on day 179, and the average concentration of cells was 7.7 × 10^7^ CFU/g (data not shown).

### 3.2. Survival of *E. coli* O157:H7 in Water Microcosms

**Figure 2:**
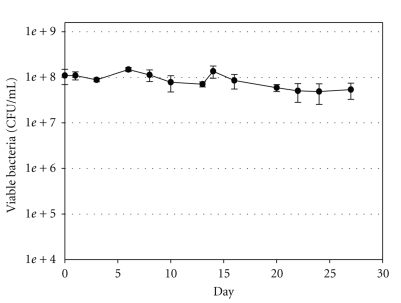
Survival of *E*. *coli *O157:H7 in sterile stream water microcosms at 15°C at 60 rpm. The average concentrations of *E*. *coli *O157:H7 were determined by the spread plate method and are shown as CFU per milliliter. Error bars represent standard deviations about the means (*n* = 4).

Sterile water was inoculated with 10 mL of 8.8 × 10^8^ CFU/mL of *E*. *coli *O157:H7 strain ATCC 35150. On day 0, the mean concentration of *E*. *coli *O157:H7 was 1.1 × 10^8^ CFU/mL. The microcosms were incubated at 15°C with gentle shaking at 60 rpm, and survival was monitored for 234 days. A decrease in the *E*. *coli *O157:H7 concentration below the postinoculation concentration was not observed until day 3 (Figure 2). The population decreased by less than 0.3 log by day 28. The final measurement was taken on day 234 (nearly 8 months following inoculation), and the mean concentration of cells was 2.98 × 10^4^ CFU/mL (data not shown). 

### 3.3. Microarray Analysis

**Figure 3:**
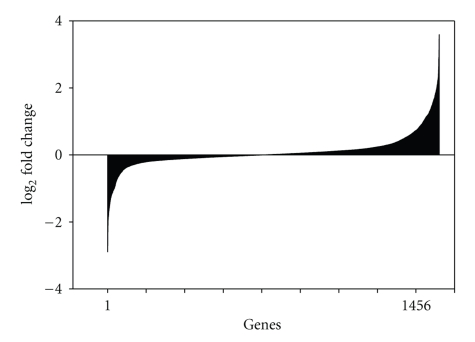
The log expression ratio of the *E*. *coli *O157:H7 genome was plotted for Luria broth versus sterile stream water. Genes more highly expressed in LB have a negative value, whereas genes more highly expressed in water have a positive value.

**Figure 4:**
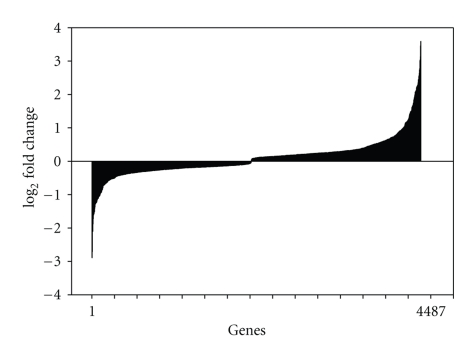
The log expression ratio of the *E*. *coli *O157:H7 genome was plotted for Luria broth versus sterile soil. Genes more highly expressed in LB have a negative value, whereas genes more highly expressed in soil have a positive value.

The genomic expression profiles of *E*. *coli *O157:H7 ATCC 35150 incubated in sterile soil, water, and LB were evaluated. The log expression ratios of the *E*. *coli *O157:H7 genome for cells grown in LB at 15°C for 48 hours with shaking at 60 rpm versus fourteen-day incubation in sterile stream water at 15°C are illustrated in Figure 3. The whole genome analysis indicated that 705 genes were more highly expressed in LB (plotted with a negative value) while 751 genes were more highly expressed in sterile stream water (plotted with a positive value) (Figure 3). Figure 4 shows the log expression ratios of the *E*. *coli *O157:H7 genome for cells grown in LB at 15°C for 48 hours versus fourteen-day incubation in sterile soil at 15°C. The analysis of the whole genome indicated that 2,664 genes were more highly expressed in LB (plotted with a negative value) while 1,823 genes were more highly expressed in sterile soil (plotted with a positive value). 

An analysis of gene ratios with significant expression levels (≥ 2 folds and *P*-value < .05) revealed that the majority of genes did not differ significantly between conditions. This was especially true of the cells grown in LB compared with the cells incubated in sterile stream water; 26 genes were more significantly expressed in LB compared to 12 genes in cells incubated in sterile stream water (Table 1). The comparison of cells incubated in sterile soil compared to cells grown in LB yielded more differences in expression; 89 genes were expressed at significantly higher levels in LB while 308 genes were more highly expressed in sterile soil (Table 2). 

A functional group analysis was performed for significantly expressed genes in LB versus sterile soil (Table 2) and LB versus sterile stream water (Table 1). Functional group analysis of LB versus sterile soil revealed that cells incubated in sterile soil expressed more genes for antibiotic resistance, biosynthesis, DNA replication/repair and restriction/modification, metabolism, pathogenesis and virulence, phages, transposons, and plasmids, ribosomal proteins, stress response, transcription, RNA processing, and degradation, translation and posttranslational modification, and transport and binding proteins (Table 2). Functional group analysis of LB versus sterile stream water revealed that cells incubated in sterile stream water expressed more genes of unknown function while cells grown in LB expressed more genes for ribosomal proteins, signaling and motility, stress response, transcription, RNA processing, and degradation, translation and posttranslational modification, and transport and binding proteins (Table 1). The nature of these differences in genomic expression is described in detail in Tables 3 and 4. For example, seven of the genes for amino acid biosynthesis were expressed at significantly higher levels in soil than in LB (Table 3). There were no differences in expression of these amino acid biosynthesis genes between cells grown in LB and cells incubated in sterile natural water (Table 4). Of the 55 genes that are known to encode ribosomal proteins, 45 were expressed at significantly higher levels in soil compared to Luria broth (Table 2). Only one ribosomal protein gene (*rpmC*) was significantly expressed in cells grown in LB compared to water (Table 1). 

The genes responsible for the stress response include those that function in temperature shock, acid tolerance, the SOS response, and osmotic challenge. Eighteen stress response genes were significantly expressed in cells incubated in sterile soil compared to LB (Table 2). On the other hand, three stress response genes were more significantly expressed in LB compared to cells incubated in sterile natural water (Table 1). The *rpoS* gene is induced in response to entry into stationary phase and also by stresses such as weak acids, starvation, osmotic challenge, and temperature changes. The expression of *rpoS *was significantly elevated in soil (2.68-fold induction) (Table 3). The *rpoH* heat shock sigma factor 32 (*σ* ^32^), which regulates the heat shock response, was more highly expressed in soil compared to LB (3.19-fold induction) (Table 3). Cells grown in soil expressed heat shock genes *dnaK *and *htpX* at significantly greater levels (Table 3). In addition, table 3 shows that *rseA*, an antisigma regulator of the *rpoE *envelope heat stress system, was induced in cells incubated in soil (3.29-fold induction). Numerous cold shock genes were significantly expressed in cells incubated in soil compared to cells grown in LB: *cspA, cspE, cspG, ymcE, deaD, yfiA *(Table 3). Only one cold shock gene (*cspC) *was expressed at significantly lower levels in cells incubated in sterile water compared to cells grown in LB (0.48-fold repression) (Table 4). Two genes involved in the SOS response were significantly expressed in cells grown in soil compared to LB: *recA *and *sulA.* This regulatory network is induced by DNA damage or interference with DNA replication. The osmotically inducible gene *osmB *was expressed at significantly higher levels in cells incubated in soil (4.77-fold induction). *osmB *encodes an outer membrane protein of unknown function. Seven genes that aid in pathogenesis and virulence were significantly expressed in cells incubated in soil compared to those grown in LB (Table 2). In particular, the *vacB *gene was expressed (2.26-fold induction) (Table 3). *tolA*, a gene involved in colicin production, was significantly expressed in cells incubated in soil compared to cells grown in LB (2.26-fold induction) (Table 3). Also, the *soxS *gene was more highly expressed in cells incubated in sterile soil microcosms (2.44-fold induction) (Table 3). Finally, three antibiotic resistance genes (*marR, marA, *and* marB*) were expressed at significantly higher levels in cells incubated in sterile soil microcosms compared to LB (Table 2).

## 4. Discussion

*E*. *coli *O157:H7 may encounter conditions that are less than optimal for growth in soil and water and must adapt to these conditions in order to survive. Various stress response mechanisms allow this pathogen to adapt to sublethal environmental conditions. Extended exposure to these stresses enables *E*. *coli *O157:H7 to survive under more severe conditions, increases its pathogenesis, and enhances its resistance to chemicals typically used in water distribution systems [14]. This has significant public health implications because *E*. *coli *O157:H7 could develop a disinfectant-resistant phenotype during transport to water treatment plants [14]. Therefore, this study investigated the survival and genetic expression profiles of *E*. *coli *O157:H7 in sterile soil and sterile natural water. Our results indicate that *E*. *coli *O157:H7 can persist for long periods of time in sterile soil and sterile stream water. In addition, we found that *E*. *coli *O157:H7 exhibits differential gene expression profiles in sterile soil and sterile stream water compared to cells freshly grown in LB. This survival does not account for the possible effects of competition with other bacteria or interactions with predatory protozoa. Under natural conditions, where predators and other bacteria are present, a net die off of *E*. *coli *O157:H7 would likely occur. It is also possible that the environmental persistence of *E*. *coli *O157:H7 cells initially grown in fecal extracts could be different.

Microarray analysis revealed that cells incubated in sterile soil for 14 days remain very active. In fact, 308 genes were found to be more highly expressed in these cells compared to cells grown in LB. A functional group analysis revealed that the majority of these genes were involved in amino acid biosynthesis, DNA replication and repair, pathogenesis and virulence, the stress response, ribosomal proteins, antibiotic resistance, transcription, and translation. On the other hand, microarray analysis of cells placed in sterile stream water for 14 days revealed that only 12 genes were more highly expressed in this condition. The majority of these genes are uncategorized and of unknown function. There was a marked difference in the expression of ribosomal protein and translation genes. Typically, faster-growing cells synthesize proteins more rapidly and contain more ribosomes [19, 20]. Tao et al. [18] studied the gene expression of *E*. *coli* K12 in response to nutrient limitation. These researchers found that 42 ribosomal protein genes were expressed at significantly higher levels in cells grown under high nutrient conditions. The present study, on the other hand, revealed that 45 ribosomal protein genes were more highly expressed in cells incubated in sterile soil compared to cells grown in LB. The exception to growth-rate-dependent regulation of ribosome number occurs at very low growth rates [21]. When *E*. *coli *cells adjust to a slow growth rate from a fast one, RNA accumulation is attenuated for a short time until the RNA content is reduced to that characteristic of cells grown at the slower rate [22]. 

It is thought that the same mechanism that functions during amino acid starvation also functions during growth rate transitions. In fact, the continued accumulation of RNA in cells under partial amino acid starvation has been shown to be accompanied by a continued synthesis in ribosomal proteins [23]. This could account for the higher expression of genes for ribosomal proteins observed in the present study as genes encoding the enzymes needed for amino acid biosynthesis were more highly expressed in cells incubated in sterile soil microcosms. In *E*. *coli, *there are 97 known genes responsible for encoding the enzymes needed for amino acid biosynthesis [18]. Previous results [18] indicate that these genes are induced for growth in low nutrient environments as appears to be the case in the present study. Growth conditions that lead to a decreased rate of ribosome synthesis typically result in an excess of ribosomal proteins, and their transcript levels are higher in faster growing cells. 

The regulatory mechanism that controls the general stress response is the RpoS sigma factor (*σ* ^38^) and is encoded by the *rpoS* gene [24]. An early adaptation in cells exposed to environmental stresses involves the expression of *rpoS*. This gene, which controls the expression of more than 50 proteins, is induced in response to entry into stationary phase and also by stresses such as weak acids, starvation, osmotic challenge, and temperature changes [13]. The expression of *rpoS *in cells in sterile soil microcosms was 2.68-fold higher when compared to cells cultured in Luria broth. In addition, the expression of 18 genes involved in the stress response was more highly expressed in cells from soil. These genes regulate cellular response to cold shock, heat shock, acid tolerance, osmotic challenge, and the SOS response [24]. This indicates that the soil environment stressed these cells, and they turned on genes to cope with sublethal environmental conditions. 

The heat shock response is a protective mechanism to cope with heat-induced damage to proteins; however, there is evidence suggesting that these genes are also induced in response to acidic conditions [17], SOS-inducing treatments [25], and sublethal exposure to chlorine [26]. Most heat shock proteins act as molecular chaperones that bind to and stabilize unfolded proteins and promote protein refolding and proper assembly [27]. This is the case with the product of the *dnaK* gene. The *dnaK *gene product has been shown to regulate other heat shock proteins, such as *htpX*, and play a major role in digesting irreversibly heat damaged polypeptides [28]. In addition to heath shock proteins, numerous cold shock genes were significantly expressed in cells incubated in soil compared to cells grown in LB: *cspA, cspE, cspG, ymcE, deaD, yfiA. *These genes protect the cell during sublethal environmental temperatures. CspA is the major cold shock protein of pathogenic *E*. *coli*. It functions as an RNA chaperone and facilitates translation at low temperatures [29]. A specific sigma factor has not yet been identified in the case of the cold shock response [27].

Two genes involved in the SOS response were significantly expressed in cells grown in soil compared to LB: *recA *and *sulA.* This regulatory network is induced by DNA damage or interference with DNA replication. The RecA protein functions as a positive control for SOS regulation, is required for all homologous recombination in *E*. *coli*, and catalyzes synapsis and strand exchange between homologous molecules [30]. The *sulA *gene product functions as an inducible inhibitor of septation [31]. When cells are exposed to SOS-inducing environments, they will continue to elongate but fail to septate and thus form filaments. 

Several genes responsible for the pathogenesis and virulence of *E*. *coli *O157:H7 were significantly expressed in cells from sterile soil microcosms. The *vacB* gene, which is required for the full expression of the virulence phenotype in *E*. *coli *[32], was highly expressed in cells incubated in soil. Moreover, a gene involved in colonic acid biosynthesis, *wcaL*, was more highly expressed in soil compared to LB. *wcaL *is the last gene of the colanic acid gene cluster [33]. Colanic acid forms a protective capsule around the bacterial cell surface and plays a role in pathogenesis [34]. Danese et al. [35] demonstrated that colonic acid synthesis is upregulated in biofilms and is not synthesized in planktonic cells under normal laboratory conditions. This may account for the differences in colonic acid gene expression observed between the cells incubated in sterile soil microcosms (in which biofilms are likely) compared to the LB control. A gene involved in colicin production,* tolA*, was significantly expressed at a higher level in cells incubated in soil compared to cells grown in luria broth. Colicins are antibacterial proteins produced by some strains of *E*. *coli* that kill competing strains of bacteria by inhibiting energy metabolism, protein synthesis, or DNA synthesis [36]. Colicins are also known to increase bacterial resistance to host defense. In addition, three genes (*marR, marA, *and* marB*) responsible for multiple antibiotic resistance were more highly expressed in sterile soil. The multiple antibiotic resistance (*mar*) locus in *E*. *coli* is composed of two operons (*marC *and *marRAB*). Expression of the *marRAB *operon protects *E*. *coli *against numerous antibiotics [37]. Moreover, the elevated expression of the *soxS* gene product has been associated with the multiple antibiotic resistance (mar) phenotype [37]. The collective expression of these genes and the genes involved in the general stress response may contribute to bacterial survival and virulence during infection. In fact, there is evidence that antibiotic treatment increases the development of hemolytic uremic syndrome (HUS) in children with *E*. *coli *O157:H7 infection [38]. 

In conclusion, Affymetrix GeneChip *E*. *coli* Genome Arrays were used to demonstrate that *E*. *coli *O157:H7 cells placed in sterile soil and water microcosms at 15°C for 14 days exhibit differential gene expression compared to cells grown in LB at 15°C for 48 hours. The cells incubated in sterile soil microcosms were undoubtedly stressed and therefore in a different physiological state than cells grown in LB at 15°C for 48 hours. These cells exhibit a phenotype that may lead to stress-associated disinfection resistance, increased pathogenesis, and virulence. This has important implications in water treatment and public health because surface and ground waters are the source for municipal drinking water. Further research on the mechanisms and regulation of the stress response of *E*. *coli *O157:H7 is needed to prevent potential risk of disease. It is also possible that the genetics expression could be different in nonsterile environments, and this needs to be investigated.

## Figures and Tables

**Table 1:** Functional groups differentially expressed between growth in LB and growth in sterile stream water.

Functional group	Total	Higher in LB	Higher in water
Whole genome	38	26	12
Antibiotic resistance	0	0	0
Biosynthesis	0	0	0
DNA replication/repair, restriction/modification	2	1	1
Metabolism	0	0	0
Pathogenesis and virulence	0	0	0
Phage, transposon, or plasmid	0	0	0
Ribosomal proteins	1	1	0
Signaling and motility	1	1	0
Stress response	3	3	0
Transcription, RNA processing, and degradation	4	4	0
Translation and posttranslational modification	1	1	0
Transport and binding proteins	7	6	1
Uncategorized	19	9	10

**Table 2:** Functional groups differentially expressed between growth in LB and growth in sterile soil.

Functional group	Total	Higher in LB	Higher in soil
Whole genome	397	89	308
Antibiotic resistance	3	0	3
Biosynthesis	21	1	20
DNA replication/repair, restriction/modification	10	2	8
Metabolism	33	15	18
Pathogenesis and virulence	7	0	7
Phage, transposon, or plasmid	8	3	5
Ribosomal proteins	45	0	45
Signaling and motility	2	2	0
Stress response	18	0	18
Transcription, RNA processing, and degradation	39	5	34
Translation and posttranslational modification	27	2	25
Transport and binding proteins	48	21	27
Uncategorized	136	38	98

**Table 3:** Selected genes differentially expressed between growth in LB and sterile soil microcosms.

Function and gene	Description	Logarithmic ratio (S/C)	*P*-value
Amino acid biosynthesis			
* hisG*	Histidine biosynthesis	2.05	1.04*E*-03
* argB*	Arginine biosynthesis	3.05	1.60*E*-04
* argC*	Arginine biosynthesis	3.52	4.13*E*-04
* argG*	Arginine biosynthesis	3.07	2.56*E*-06
* asnB*	Asparagine synthetase B	3.41	1.66*E*-02
* thrL*	Thr operon leader peptide	3.43	1.23*E*-03
* cysK*	Cysteine biosynthesis	3.53	2.00*E*-06

Antibiotic resistance			
* marA*	Multiple antibiotic resistance protein	4.20	2.31*E*-09
* marB*	Multiple antibiotic resistance protein	4.41	3.25*E*-05
* marR*	Multiple antibiotic resistance protein	5.16	2.51*E*-07

DNA replication/repair, restriction/modification			
* priB*	Primosomal replication protein	2.19	1.23*E*-03
* topA*	DNA topoisomerase I	2.33	6.56*E*-08
* fis*	DNA binding protein Fis	2.37	3.72*E*-05
* priA*	Primosome assembly protein	2.60	4.67*E*-02

Metabolism			
* aceE*	Pyruvate dehydrogenase E1 subunit	2.03	5.14*E*-06
* asmA*	Protein asmA precursor; electron transport	2.21	3.40*E*-02
* lpdA*	Dihydrolipoamide dehydrogenase; energy metabolism	2.24	1.16*E*-04
* nemA*	N-ethylmaleimide reductase; central intermediary metabolism	2.71	1.56*E*-05
* glpC*	Anaerobic glycerol-3-phosphate dehydrogenase subunit C	2.81	7.83*E*-03
* yfhO*	Cysteine metabolism; amino acid metabolism	3.25	3.64*E*-05
* icdA*	Isocitrate dehydrogenase; TCA cycle metabolism	3.48	9.13*E*-05
* adhC*	Alcohol dehydrogenase class III; energy metabolism	4.19	9.70*E*-05
* yibO*	Phosphoglyceromutase; carbohydrate metabolism	6.21	8.10*E*-03
* ttdB*	L(+)-tartrate dehydrase; energy metabolism	0.44	2.22*E*-06
* hycG*	Formate hydrogenlyase subunit 7; mitochondrial electron transport	0.44	3.00*E*-06
* phnH*	Carbon-phosphorus lyase complex subunit; central intermediary metabolism	0.45	2.51*E*-04
* mhpF*	Acetaldehyde dehydrogenase; amino acid metabolism	0.46	1.45*E*-06
* phnJ*	Carbon-phosphorus lyase complex subunit; central intermediary metabolism	0.46	5.55*E*-05
* hycF*	Hydrogenase 4 Fe-S subunit formate hydrogenlyase, complex iron-sulfur protein	0.47	2.96*E*-05
* eutI*	Phosphate acetyltransferase	0.47	8.02*E*-04
* ygjL*	2,4-dienoyl-CoA reductase (NADPH), NADH and FMN-linked	0.49	4.90*E*-01

Pathogenesis and virulence			
* vacB*	Ribonuclease R, exoribonuclease R, RNase R	2.26	1.44*E*-05
* tolA*	Colicin production	2.26	2.51*E*-05
* wcaL*	Colanic acid biosynthesis; resistance to acid stress, desiccation, and thermal stress	2.51	1.32*E*-02
* ygeO*	Hypothetical protein	2.66	1.04*E*-02

Phage, transposon, or plasmid			
* sieB*	Rac prophage; phage superinfection exclusion protein	0.43	5.93*E*-03
* Lar*	Rac prophage; restriction alleviation protein	0.43	9.19*E*-03
* ydaE*	Rac prophage; conserved protein	0.44	2.28*E*-03
* ydaQ*	Rac prophase; conserved protein	0.45	2.22*E*-03
* ydaC*	Rac prophage predicted protein	0.50	8.85*E*-03
* ymfP*	E14 prophage; conserved protein	4.46	1.87*E*-02
* mprA*	Translational repressor mprA; plasmid related function	3.53	1.78*E*-05

Ribosomal proteins			
* rpsU*	30S ribosomal protein S21	2.25	1.12*E*-04
* rpsT*	30S ribosomal protein S20	2.35	4.46*E*-04
* rplY*	50S ribosomal protein L25	2.35	2.21*E*-03
* rpmF*	50S ribosomal protein L32	2.43	1.16*E*-03
* rpmH*	50S ribosomal protein L34	2.52	3.30*E*-05
* rplK*	50S ribosomal protein L11	2.67	9.18*E*-04
* rpsR*	30S ribosomal protein S18	2.87	7.21*E*-05
* rplA*	50S ribosomal protein L1	2.87	9.28*E*-05
* rpmD*	50S ribosomal protein L30	3.17	3.14*E*-05
* rpsJ*	30S ribosomal protein S10	3.24	6.19*E*-04
* rpmE*	50S ribosomal protein L31	3.32	4.52*E*-05
* rplW*	50S ribosomal protein L23	3.53	1.57*E*-04
* rpsH*	30S ribosomal protein S8	4.34	3.27*E*-04
* rplF*	50S ribosomal protein L6	4.57	1.39*E*-04
* rplX*	50S ribosomal protein L24	4.66	2.51*E*-05
* rpsN*	30S ribosomal protein S14	5.68	6.06*E*-05

Stress response			
* cspE*	Cold shock protein E	2.02	5.08*E*-03
* yfiA*	Cold shock protein, associated with 30S ribosomal subunit	2.10	1.23*E*-03
* htpX*	Heat shock protein	2.10	2.02*E*-04
* sulA*	SOS cell division inhibitor	2.17	1.69*E*-05
* ymcE*	Cold shock gene	2.33	1.49*E*-04
* dnaK *	Heat shock protein; molecular chaperone	2.48	3.21*E*-04
* recA*	Recombinase A; SOS response	2.59	8.09*E*-04
* rpoS*	Response to organic acid stress and acetate induced acid tolerance; regulatory function	2.68	6.83*E*-04
* ahpC*	Alkyl hydroperoxide reductase C22 protein; oxidative stress	2.91	9.08*E*-06
* dead*	Cold shock DEAD box protein A	3.04	1.18*E*-05
* rpoH*	RNA polymerase sigma factor; heat response	3.19	5.63*E*-06
* Spy*	Envelope stress induced periplasmic protein	3.54	6.83*E*-08
* osmB*	Osmotic adaptation; Osmotically inducible lipoprotein B precursor	4.77	1.81*E*-04
* cspG*	Cold shock protein	5.04	2.93*E*-05
* cpxP*	Envelope stress response	7.08	1.17*E*-05
* cspA*	Cold shock protein cspA, major cold shock protein	8.59	6.77*E*-05

Transcription, RNA processing, and degradation			
* rpoB*	DNA directed RNA polymerase beta subunit; transcription	2.12	1.54*E*-04
* rpoD*	Hypothetical protein, RNA polymerase sigma factor	2.20	1.79*E*-03
* Pnp*	Polyribonucleotide, nucleotidyltransferase; RNA processing	2.21	1.58*E*-03
* rpnA*	Ribonuclease P	2.31	4.01*E*-05
* Rne*	Ribonuclease E, fused ribonuclease E: endoribonuclease	2.72	1.33*E*-03
* rpoA*	DNA-directed RNA polymerase alpha subunit; transcription	2.92	1.72*E*-04
* nusA*	Transcription elongation factor NusA	3.16	1.15*E*-04
* Rho*	Transcription termination factor Rho	3.34	5.92*E*-05

Translation and posttranslational modification			
* soxS*	Regulatory protein, DNA binding dual transcriptional regulator	2.44	2.47*E*-04
* tufA*	Elongation factor Tu, protein chain elongation factor (EF-Tu)	2.61	8.28*E*-05
* infA*	Translation initiation factor IF-1	3.09	4.37*E*-05
* infC*	Translation initiation factor IF-3	3.31	3.27*E*-05
* fusA*	Elongation factor EF-2	3.36	7.54*E*-05
* infB*	Translation initiation factor IF-2	4.13	5.84*E*-04

Transport and binding proteins			
* phnO*	Phosphonate transport, N-acetyltransferase activity	0.46	1.40*E*-06
* treB*	PTS system, trehalose-specific IIBC component; transport of small molecules	0.47	3.04*E*-04
* malK*	Maltose/maltodextrin transport	0.48	1.49*E*-05
* thiQ*	Thiamine transport	0.49	1.48*E*-04
* fepB*	Iron-enterobactin transporter subunit	0.49	7.73*E*-05
* cusB*	Copper efflux system protein	2.02	4.55*E*-06
* yjbB*	Phosphate transport, sodium dependent phosphate transporter	2.07	5.77*E*-03
* livJ*	Leucine/isoleucine/valine transporter subunit	2.22	3.16*E*-04
* oppA*	Oligopeptide transporter subunit	2.32	2.98*E*-04
* lolE*	Lipoprotein releasing system, transmembrane protein lolE	2.32	1.54*E*-02
* prlA*	Preprotein translocase; protein transport	2.46	3.03*E*-05
* glnH*	Glutamine ABC transporter, periplasmic-binding protein	2.50	5.52*E*-05
* artP*	Arginine transport	2.52	1.33*E*-05
* fepD*	Ferric enterobactin transport system	3.25	3.01*E*-02
* fepE*	Ferric enterobactin transport protein	3.85	5.61*E*-03

**Table 4:** Selected genes differentially expressed between growth in LB and sterile water microcosms.

Function and gene	Description	Logarithmic ratio (W/C)	*P*-value
DNA replication/repair, restriction/modification			
* ycbY*	DNA restriction-modification system; DNA methylation	2.38	4.13*E*-02
Membrane			
* ompA*	Outer membrane protein	0.42	4.62*E*-04
* ompX*	Outer membrane protein X; integral to outer membrane	0.43	1.09*E*-04
Metabolism			
* yfiD*	Protein yfiD, pyruvate formate lyase subunit	0.48	3.74*E*-03

Ribosomal proteins			
* rpmC*	Protein biosynthesis, structural constituent of ribosome, intracellular ribosome, ribonucleoprotein complex	0.48	3.25*E*-04

Regulatory RNA			
* gcvB*	Regulatory sRNA	0.23	1.00*E*-03
* csrC*	Regulatory RNA	0.39	4.40*E*-03
* vmicF*	Regulatory sRNA	0.41	1.99*E*-03
* ryhA*	Unknown RNA	0.46	8.79*E*-03
Stress response			
* cspC*	Cold-shock stress protein	0.48	1.41*E*-04
* dps*	DNA protection during starvation conditions	0.49	3.75*E*-04

Transcription, RNA processing, and degradation			
* himA*	Integration host factor alpha subunit; DNA recombination and transcription regulation	0.49	9.13*E*-03

Transport and binding proteins			
* ompF*	Outer membrane protein F precursor; ion transport, porin activity	0.46	1.21*E*-03
* potF*	Putrescine-binding, periplasmic protein precursor	2.71	3.22*E*-02
